# Towards a New Era

**Published:** 1920-02-07

**Authors:** 


### THE PUBLIC WELL-BEING.

### TOWARDS A NEW ERA.

### International Safeguards for Women and Children.

It is not outside the considerations to which these pages are devoted to give attention to international social and industrial conditions which have been under discussion recently. International labour conferences entered upon a new era with the first meeting held under the Peace Treaty at Washington last autumn and continued a few days ago in Paris. It was really the first comprehensive attempt to improve the social and industrial circumstances of the world, and a number of vitally important principles were agreed to by East and West. It was not an isolated effort, but the start of a sustained and continuous attempt organised for what might be termed domestic welfare in every land.

As a result of this first conference, six draft conventions are to be presented for ratification by the parliaments of the forty States who are members. They have been summarised as follows :—

(1) The daily hours of work in industry to be not more than eight, with a forty-eight hours' week. Where the hours on one day are less than eight, the limit may be exceeded on other days, provided that they are never more than nine. In shifts, the limit may be exceeded, provided the average number of working hours over a period of three weeks does not exceed fifty-six a week. Some other exceptions of the same nature are made. In Japan there will be a limit of fifty-seven hours for workers over fifteen, with sixty hours for the silk industry, and in India sixty hours for miners and certain railway workers. In Greece and Rumania the above rules are not applicable till 1924.

(2) Children under fourteen are not admitted to industry. In Japan and India, in certain trades children under twelve are barred.

(3) Each country shall establish free employment agencies, and furnish information to the International Labour Office every three months.

(4) Women are not to be employed between 10 P.M. and 5 A.M.

(5) Persons under eighteen are not to be employed between 10 P.M. and 5 A.M. For Japan the limit is fifteen till 1925, and for India the limit is fourteen.

(6) No women to work for six weeks after childbirth, and all women to have the right to cease work six weeks before. Maintenance by the State while out of work owing to childbirth.

The provisions to safeguard the health of women and children workers, and to limit the age at which the latter may be employed are particularly attractive. There are many grave abuses in this connection in England, where we justly pride ourselves on our superior conditions, but in Japan and some of the Eastern lands the conditions are little short of slavery. It is scarcely too much to say that the whole peace, happiness, and health of the world depend as much upon the proper regulation of work, adequate remuneration, and reasonable facilities for leisure, as upon schemes for disarmament, alliances, and all the rest of the things that absorb the minds of politicians. The sooner this is realised and acted upon the better will it be. It would be interesting if we could have some statistics upon one point alone—namely, the average length of rest that the working woman is able to take at times of childbirth. When all the hazards of infant life are considered, the wonder is, not that infant mortality is so high, but that it is not higher.

If the International Labour Conference maintains its high promise, and the social and other organisations at the opposite end of the scale continue their efforts in their prescribed areas, whether in parish, village, town, or country, there will be renewed hope that the lead from the international body and the steady detailed practice of the local organisations may leaven the whole lump, and gradually make for better things.

